# Crystal Structure of Arginine Methyltransferase 6 from *Trypanosoma brucei*

**DOI:** 10.1371/journal.pone.0087267

**Published:** 2014-02-03

**Authors:** Chongyuan Wang, Yuwei Zhu, Jiajia Chen, Xu Li, Junhui Peng, Jiajing Chen, Yang Zou, Zhiyong Zhang, Hong Jin, Pengyuan Yang, Jihui Wu, Liwen Niu, Qingguo Gong, Maikun Teng, Yunyu Shi

**Affiliations:** 1 Hefei National Laboratory for Physical Sciences at Microscale and School of Life Sciences, University of Science and Technology of China, Anhui, China; 2 Department of Chemistry and Institute of Biomedical Science, Fudan University, Shanghai, China; University of Alabama at Birmingham, United States of America

## Abstract

Arginine methylation plays vital roles in the cellular functions of the protozoan *Trypanosoma brucei*. The *T. brucei* arginine methyltransferase 6 (TbPRMT6) is a type I arginine methyltransferase homologous to human PRMT6. In this study, we report the crystal structures of apo-TbPRMT6 and its complex with the reaction product S-adenosyl-homocysteine (SAH). The structure of apo-TbPRMT6 displays several features that are different from those of type I PRMTs that were structurally characterized previously, including four stretches of insertion, the absence of strand β15, and a distinct dimerization arm. The comparison of the apo-TbPRMT6 and SAH-TbPRMT6 structures revealed the fine rearrangements in the active site upon SAH binding. The isothermal titration calorimetry results demonstrated that SAH binding greatly increases the affinity of TbPRMT6 to a substrate peptide derived from bovine histone H4. The western blotting and mass spectrometry results revealed that TbPRMT6 methylates bovine histone H4 tail at arginine 3 but cannot methylate several *T. brucei* histone tails. In summary, our results highlight the structural differences between TbPRMT6 and other type I PRMTs and reveal that the active site rearrangement upon SAH binding is important for the substrate binding of TbPRMT6.

## Introduction

Protein arginine methylation is a widespread post-translational modification that plays important roles in various processes, such as transcriptional regulation, RNA processing, DNA repair, and signal transduction [1]–[3]. The set of protein arginine methyltransferases (PRMTs) is a family of enzymes that catalyze the transfer of a methyl group from S-adenosyl-L-methionine (SAM) to the guanidino nitrogen of an arginyl residue to produce S-adenosyl-L-homo-cysteine (SAH) and methyl arginyl residues. Based on the methyl arginine products, PRMTs are primarily classified into three types. Type I and II PRMTs both catalyze ω-N^G^-mono-methylarginine (MMA) in the first step; type I PRMTs subsequently produce asymmetric N^G^, N^G^-dimethylarginine (aDMA), whereas type II PRMTs generate symmetric N^G^, N’^G^-dimethylarginine (sDMA). Type III PRMTs only catalyze MMA [2]. Eleven human PRMTs have been identified: PRMT1, −2, −3, −4 (CARM1), −6, and −8 with type I enzyme activities, PRMT5 and −9 with type II enzyme activities, PRMT7 with type III activity, and PRMT10 and PRMT11, the activities of which have not yet been characterized [3].

Human PRMT6 (HsPRMT6) exclusively localizes in the nucleus [4], and this localization is correlated with its function in DNA repair and transcriptional regulation [5]–[7]. HsPRMT6 methylates a few substrates, including HMG1A [8]–[10], DNA polymerase beta [5], tumor repressor p16 [11], histones [12]–[14], and several HIV proteins [15]–[16].

*Trypanosoma brucei,* the protozoan parasite that causes African sleeping sickness, owns five putative PRMTs in its genome [17], and four of these have been characterized: TbPRMT1 and TbPRMT6 with type I activity [18]–[19], TbPRMT5 with type II activity [20], and TbPRMT7 with type III activity [21]. More than 850 arginine-methylated proteins have been identified in *T. brucei*, and their functions range from RNA processing, DNA replication and repair, and signal transduction to metabolism [22]–[23], suggesting the vital roles of arginine methylation in the cellular functions of *T. brucei*.

TbPRMT6 is the homologue of HsPRMT6 in *T. brucei* with 31% amino acid identity. TbPRMT6 also has homologues in the related kinetoplastid parasites *T. cruzi* and *Leishmania major* with 57% and 47% identity, respectively [19]. TbPRMT6 lacks the N-terminal nuclear localization signal (NLS) peptide present in HsPRMT6 and almost exclusively localizes in the cytoplasm with a slight degree of nuclear localization [19]. Unlike other *T. brucei* PRMTs characterized to date, which methylate a wide range of substrates, TbPRMT6 displays a relatively narrower substrate range; in fact, the only known substrates of TbPRMT6 are bovine histone H3, H4, and itself [19]. The depletion of TbPRMT1, TbPRMT5, or TbPRMT7 has no effect on growth [17], [21]–[22], but the knockdown of TbPRMT6 leads to a decrease in the growth rate, indicating that TbPRMT6 plays an irreplaceable role in cellular growth. The depletion of TbPRMT6 also results in a defect in cell division, the development of a hydra morphology in procyclic-form cells, and giant rounded cells in bloodstream-form cells [19].

To investigate the structural basis for the unique properties of TbPRMT6, we report the crystal structures of apo-TbPRMT6 and its complex with the methylation product SAH (SAH-TbPRMT6); these structures were refined at 2.20 Å and 2.35 Å, respectively. The structures of TbPRMT6 highlight several structural features that are distinct from those found in previously characterized type I PRMTs, including four stretches of insertion, the absence of the β15 strand, and a unique dimerization arm. The comparison of the apo-TbPRMT6 and SAH-TbPRMT6 structures revealed the fine rearrangements of the TbPRMT6 active site upon SAH binding, which is critical for substrate binding, as demonstrated by an ITC assay. The western blotting and mass spectrometry results revealed that TbPRMT6 asymmetrically methylates bovine histone H4 tail at arginine 3 but does not methylate several peptides derived from *T. brucei* histone tails, thereby indicating its unique substrate range.

## Materials and Methods

### Cloning, Protein Expression, Purification and Peptide Synthesis

The gene encoding TbPRMT6 was amplified by PCR from the *T. brucei* cDNA library using the following primers: TbPRMT6-5′-NdeI (5′-GGGAATTCCATATGGAGTCCGGGG GGTTTG-3′) and TbPRMT6-3′-SalI (5′-TACGCGTCGACTTATTTTAACTCGAGCTCAAT GGT -3′). The PCR product was then cloned into a modified pET28a vector with a TEV protease cleavage site after the 6×His tag. The 21 N-terminal residues (SGRGKGGKGLGKGGAKRHRKV) derived from bovine histone H4 were fused to the C terminus of a thioredoxin tag and cloned into a pET28a vector (Trx-H4tail). Mutants were generated using a MutanBEST kit (Takara) and verified by DNA sequencing. All of the proteins were expressed in *E. coli* BL21 (DE3). The bacteria were cultured in LB medium at 37°C to OD600 = 0.8, shifted to 16°C, and induced with 0.5 mM IPTG for 24 h. These His-tagged proteins were purified using Ni-chelating resin (Qiagen) in 30 mM Tris buffer (pH 8.0) with 500 mM NaCl, cleaved by TEV protease overnight at 16°C to remove the His tag, and subjected to Mono Q and Superdex 200 (GE Healthcare) columns. The peptide corresponding to the first 21 residues of bovine histone H4 (AcH4-21, purity >95%, verified by Mass Spectrometry) was synthesized by GL Biochem (Shanghai).

### In vitro Methylation Assay

The *in vitro* methylation reactions contained 1 µg of Trx-H4tail and different amounts of enzymes in 30 mM Tris buffer (pH 8.0) with 100 mM NaCl, 1 mM EDTA, 5 mM DTT, and 40 µM SAM (Sigma). The reactions were performed at 37°C for 12 h and were terminated by the addition of an equal volume of loading buffer. The proteins were then boiled at 100°C for 10 min and separated by SDS-PAGE. The proteins were transferred from the gels to nitrocellulose filter membranes (Amersham) and detected by antibodies specific for bovine histone H4 asymmetric dimethyl Arg3 (39705, Active Motif).

### Isothermal Titration Calorimetry

ITC assays were conducted on a MicroCal iTC200 calorimeter (GE Healthcare) at 25°C. The concentration of TbPRMT6 was determined photometrically to 100 µM. The concentration of bovine histone H4 peptide was adjusted to 3 mM. The buffer for the proteins and peptide contained 30 mM Tris (pH 8.0), 100 mM NaCl, and 1 mM EDTA. A reference measurement (peptide injected into the buffer) was performed to compensate for the heat associated with the dilution of the peptide. Curve fitting to a one-binding-site model was performed using the ITC data analysis module of Origin 7.5 (Origin Lab Corporation) provided by the MicroCal iTC200 calorimeter.

### Mass Spectroscopy

Two micrograms of each peptide derived from the N-terminal tails of the *T. brucei* histone were incubated with 3 µg of TbPRMT6 in the buffer [30 mM Tris-HCl (pH 8.0), 50 mM NaCl, 1 mM EDTA, 10 mM DTT, and 40 µM SAM] at 37°C for 6 h, and the reactions were stopped by the addition of 10 µL of 50% TCA (v/v). The reaction mixtures were centrifuged at 14,000 g for 20 min to remove the protein and then subjected to assisted laser desorption-ionization time-of-flight mass spectrometry (MALDI-TOF MS) analysis.

### Small-angle X-ray Scattering

SAXS experiments were performed at NAL (Argonne National Laboratory). The data were acquired from three concentrations of FL-TbPRMT6: 1.0, 3.0, and 5.0 mg/ml in 30 mM Tris buffer (pH 8.0) with 200 mM NaCl and 1 mM EDTA. The data were analyzed using the ATSAS package [24] following the standard procedures. After subtracting buffer scattering, the data curves obtained from the different concentrations were scaled and merged using PRIMUS [25]. GNOM [26] was employed to estimate the particle maximum dimension (D_max_) and calculate the pair distance distribution function (PDDF). The radius of gyration R_g_ of the protein was derived in real space using the PDDF. The solute molecular mass was evaluated by the SAXSMOW online tool [27]. Models of chain-compatible dummy residues (DR) were constructed *ab initio* using the GASBOR program [28]. The resolved X-ray structure of the TbPRMT6 dimer was superimposed onto the DR model by SUPCOMB [29].

### Crystallization, Data Collection, and Structure Determination

The proteins were concentrated to approximately 15 mg/ml (determined photometrically) in 10 mM Tris (pH 8.0), 200 mM NaCl, 1 mM EDTA, and 1 mM DTT. The protein-SAH complex was prepared by mixing the protein with a three-fold molecular excess of adenosyl-L-homocysteine (Sigma). All of the crystals were grown at 293K via the hanging drop method with the mother liquor containing 100 mM citrate (pH 5.5) and 20% PEG3000. The X-ray diffraction data for the crystals were collected on beamline 17U1 at the Shanghai Synchrotron Radiation Facility (SSRF). The data were processed and scaled with HKL2000. The statistics of the diffraction data are summarized in Table 1. The structure of SAH-TbPRMT6 was determined through the single-wavelength anomalous dispersion (SAD) phasing technique with the iodine anomalous signal using the phenix.solve program implemented in PHENIX [30]–[31]. The initial model was built automatically using the program Autobuild in PHENIX. Using the SAH-TbPRMT6 structure as the search model, the structures of apo-TbPRMT6 were determined through the molecular replacement method using the program MOLREP implemented in CCP4i [32]–[33]. All of the initial models were refined using the maximum likelihood method implemented in REFMAC5 [34] as part of the CCP4i program suite and rebuilt interactively using the program COOT [35]. During the later stage, the restrained positional and B-factor refinement was performed using the program phenix.refine during the refinement. The final models were evaluated with the programs MOLPROBITY [36] and PROCHECK [37]. The crystallographic parameters are listed in Table 1. All of the structures in the figures were prepared with PyMOL [38].

**Table 1:** Data Collection and Refinement Statistics for apo- TbPRMT6 and TbPRMT6-KI-SAH.

Data collection statistics	TbPRMT6-KI-SAH	apo- TbPRMT6
Space Group	C2221	P21
Unit Cell Parameters		
a, b, c (Å)	1014,1085,1379	739, 1433, 756
α, β, γ (°)	90, 90, 90	90, 967, 90
Wavelength(Å)	154	09792
A Resolution limits(Å)	5000-235(239-235)	5000-220(224-220)
No of unique reflections	31830	77167
Completeness (%)	999(999)	981(973)
Redundancy	144(142)	38(37)
BRmerge (%)	109(434)	0075(0578)
Mean I/σ(I)	289(645)	168(30)
Refinement Statistics		
Resolution limits(Å )	50-235	4400-220
CRwork(%)/DRfree(%)	2044/2493	2020/2487
Rmsd for bonds (Å)	0009	0008
Rmsd for angles (°)	1198	1139
B factor (Å^2^)		
Protein	285	377
SAH	242	
Water	250	323
No of non-hydrogen protein atoms	5258	10380
No of water oxygen atoms	57	162
Ramachandran plot (%)		
most favored regions	922	919
additional allowed regions	74	75
generously allowed regions	03	07
PDB entry	4LWP	4LWO

A Values in parentheses are for the highest resolution shell.

B Rmerge = Σh Σl |Ihl- <Ih>|/ Σh Σl <Ih>, where Ihl is the lth observation of reflection h and <Ih> is the weighted average intensity for all observations l of reflection h.

C Rwork factor = Σh||Fobs(h) |- |Fcal(h) ||/Σh|Fobs(h) |, where Fobs(h) and Fcal(h) are the observed and calculated structure factors for reflection h respectively.

D Rfree factor was calculated same as Rwork factor using the 5% the reflections which were selected randomly and omitted from refinement.

## Results and Discussion

### Overall Structure

**Figure 1:**
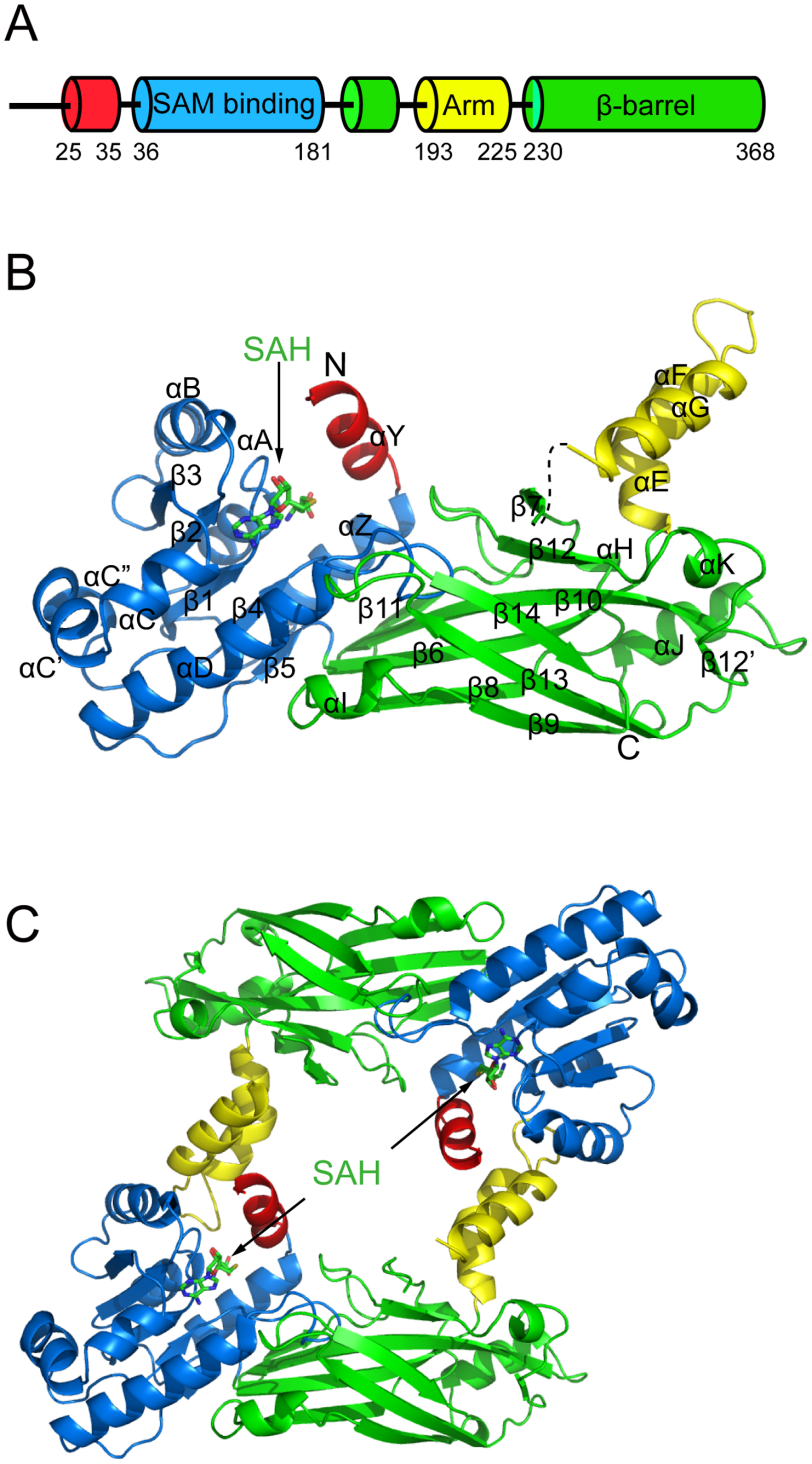
Structure of TbPRMT6. (A) Schematic diagram of the domain arrangement of TbPRMT6 (B) Overall structure of a monomer The N-terminal helix αY, the SAM-binding domain, the dimerization arm, and the β-barrel domain are shown in red, blue, yellow, and green, respectively The cofactor SAH is shown in the stick model The segment between helix αG and strand β7 is invisible and is shown as a dashed line (C) Structure of the TbPRMT6 dimer.

The full-length TbPRMT6 was expressed, purified, and crystallized. Structures of apo-TbPRMT6 and in complex with SAH (SAH-TbPRMT6) were resolved and refined at 2.20 Å and 2.35 Å, respectively (Table 1). We primarily describe the structure of SAH-TbPRMT6 unless otherwise noted. The overall monomeric structure of TbPRMT6 consists of three parts: a SAM-binding domain (residues 36–181, blue), a dimerization arm (residues 193–225, yellow), and a β-barrel domain (residues 182–192 and residues 230–368, green) (Figure 1A and Figure 1B). The SAM-binding domain contains a Rossmann fold with three additional helices (αC, αC’, and αC”) inserted between strands β3 and β4 (Figure 1B). The dimerization arm, composed of three helices (αE, αG, and αF), inserts into the β-barrel domain and divides it into two parts (residues 182–192 and residues 230–368) (Figure 1A and Figure 1B). The SAM-binding domain and β-barrel domain are connected by a cis-proline (P181), which is strictly conserved in all known PRMTs. The N terminus (residues 1–24) and the segment between strand β6 and helix αE (residues 228 and 229) are too flexible to be observed in the electron density map.

### SAH Recognition

**Figure 2:**
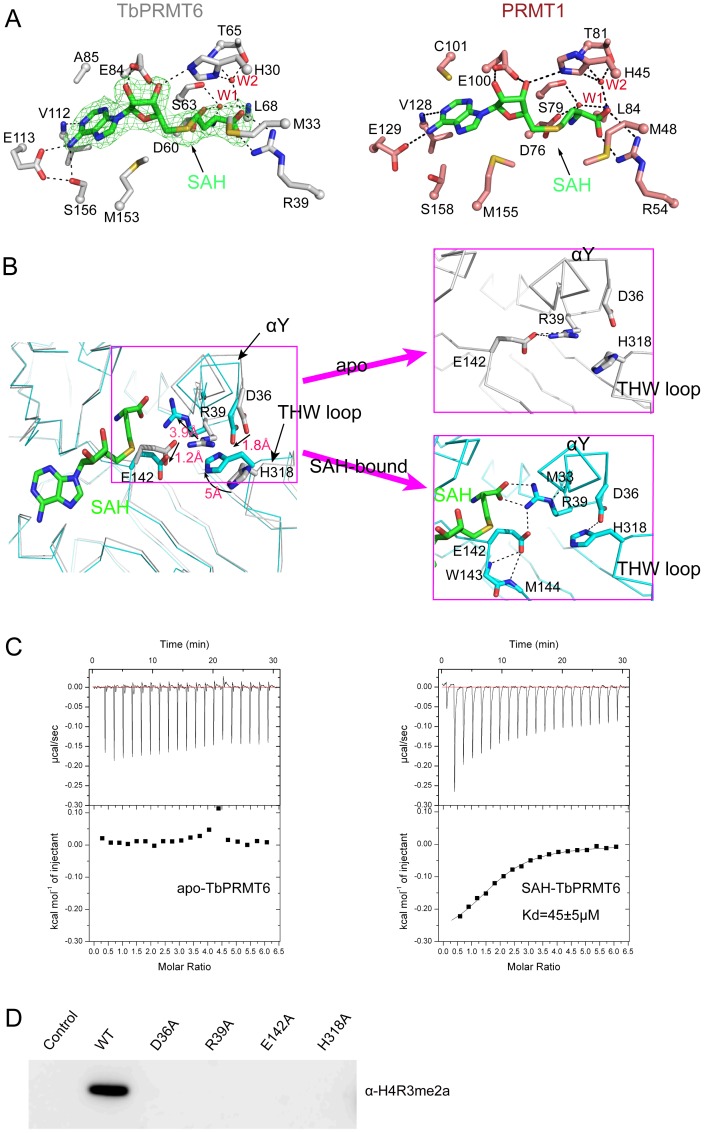
SAH recognition. (A) SAH interaction with TbPRMT6 (left) and rat PRMT1 (right) The residues are shown in the stick model and labeled, and the water molecules are shown as red spheres and labeled with W1 and W2 A representative omit (Fo-Fc) electron density map (green) shows the bound SAH The dashed lines represent hydrogen bonds (B) The conformational change in the active site of TbPRMT6 induced by SAH binding The left figure is the superposition of the apo (grey) and SAH-bound (cyan) structures of TbPRMT6 The movements of the key residues are highlighted by arrows and distances The right figures are enlarged views of the active site of TbPRMT6 in the free and SAH-bound states The dashed lines represent hydrogen bonds (C) ITC-based measurements of the bindings of AcH4-21 to apo and SAH-bound TbPRMT6 The fitted *K_d_* of AcH4-21 to SAH-bound TbPRMT6, including the standard errors in the measurements, are indicated in the panel (D) Enzymatic assays of TbPRMT6 mutants with mutations in the residues that undergo significant rearrangement upon SAH binding.

**Figure 3:**
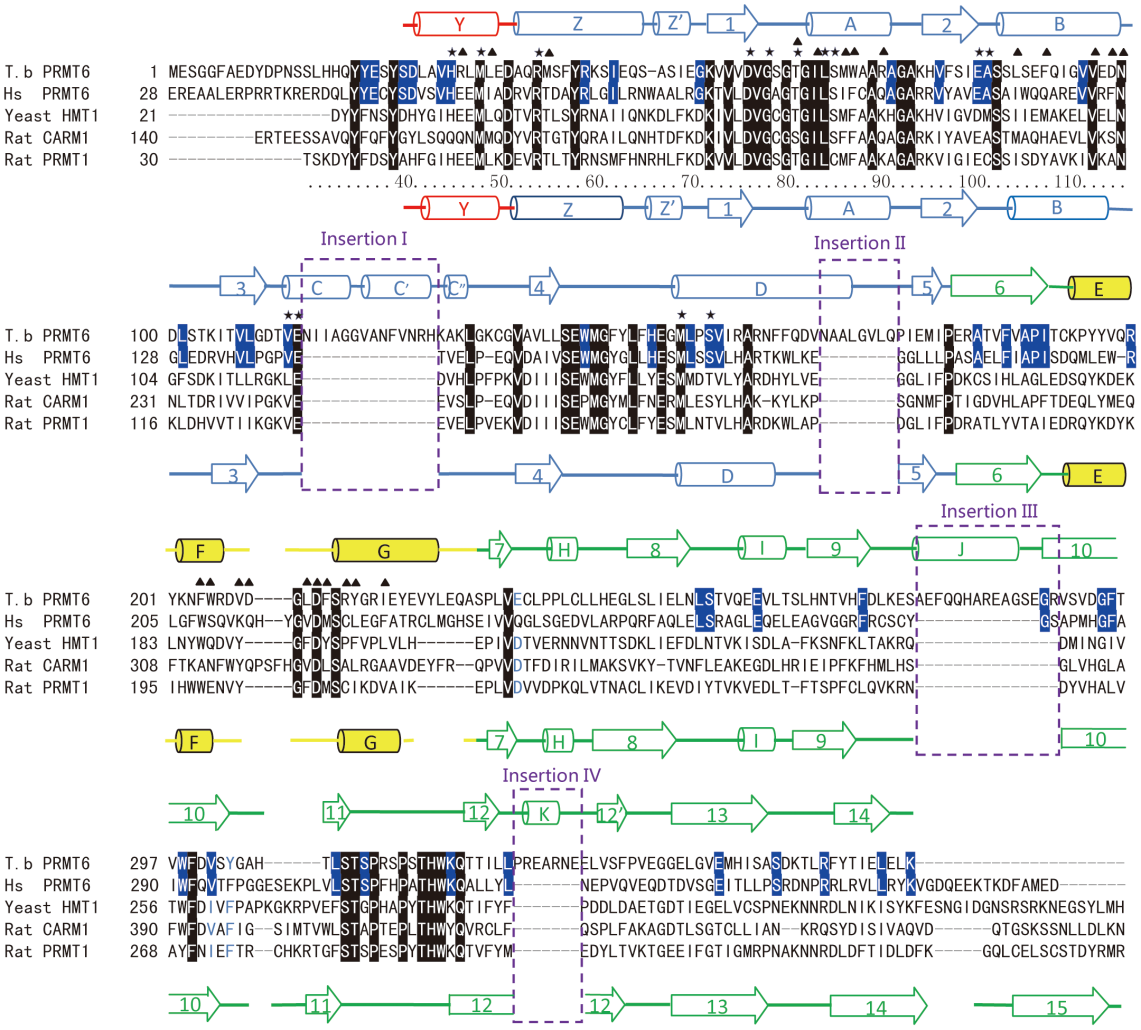
Sequence alignment of TbPRMT6, human PRMT6, rat PRMT1, and rat CARM1. The secondary structural elements of TbPRMT6 and rat PRMT1 are shown on the top of and underneath the sequence, respectively The color of the secondary structural elements is the same as in Figure 1B The residues conserved among the four enzymes are highlighted in black, and the residues conserved in the PRMT6 paralogs are highlighted in blue The asterisks and triangles above the sequence indicate the residues in TbPRMT6 that are involved in SAH recognition and dimerization, respectively The four stretches of insertions are bracketed in purple dashed frames and labeled.

The SAH molecule is enveloped in the deep pocket of the SAM-binding domain formed by the helixes αY, αZ, αA, αB, αC, and αD, and the strands β2, β3, and β4 (Figure 1B). The recognition of SAH can be grouped into three moieties (Figure 2A). (i) For the adenine ring moiety, the main-chain amide of V112 (helix αC) interacts with nitrogen N1, and the carboxyl group E113 (helix αC) and the hydroxyl group of S156 (helix αD) make a hydrogen-bond network with the amino group of the adenine ring of SAH. In addition to the hydrogen bonding, the hydrophobic side chains of A85, V112 (helix C1), and M153 (helix αD) also create van der Waals contacts with the adenine ring. (ii) For the ribose moiety, the carboxyl group of E84 (strand β2) and the imidazole ring of H30 (helix αY) create hydrogen bonds with the two ribose hydroxyls. (iii) For the methionine moiety, the carboxyl group of D60 (strand β1), the carbonyl group of S63, and the backbone amide of L68 (helix αA) interact with the amino group of the homocysteine via a water molecule (labeled with W1 in Figure 2A). The guanidino group of R39 (helix αZ) makes hydrogen bonds with the carboxyl group of the homocysteine moiety, whereas the side chain of H30 (helix αY) and the hydroxyl group and backbone amide of T65 interact with the carboxyl group of the homocysteine moiety via another water molecule (labeled with W2 in Figure 2A). Furthermore, M33 in helix αY creates a van der Waals contact with the methionine moiety of the homocysteine. It is worth noting that these residues that participate in SAH recognition are very similar to those of rat PRMT1 (Figure 2A) and are highly conserved among type I PRMTs (Figure 3).

### Rearrangement of the Active Site upon SAH Binding

The superimposition of the apo-TbPRMT6 structure with the SAH-TbPRMT6 structure revealed that the active site undergoes rearrangements upon SAH binding (Figure 2B). Unlike the significant backbone motions observed in CARM1 and DOT1L active sites [39]–[40], cofactor binding primarily induced side-chain rearrangements in TbPRMT6 active site. Without SAH, the guanidine group of R39 of helix αZ and the imidazole ring of H318 of the THW loop point out from the active site. Upon SAH binding, the guanidine group of R39 moves inwards ∼3.9 Å, forming a hydrogen bond network with the carboxyl group of SAH, the backbone carbonyl of M33 of helix αY, and the carboxyl group of E142. The carboxyl group of E142 swings ∼1.8 Å to create a hydrogen bond with the backbone amide of M144. The imidazole ring of H318 swings ∼5 Å to make a hydrogen bond with the carboxyl group of D36 of helix αY, and this hydrogen bonding interaction induces a 1.8-Å movement of the carboxyl group of D36 (Figure 2B). These residues involved in the rearrangement are strictly conserved among all known type I PRMTs (Figure 3), indicating that these rearrangements likely exist in other type I PRMTs. SAH binding appeared to fix the active site residues of TbPRMT6 in conformations that are favorable for substrate binding. To test this hypothesis, we analyzed the affinity of AcH4-21 (first 21 residues of bovine histone H4, a widely used *in vitro* substrate for PRMTs) to apo-TbPRMT6 and SAH-TbPRMT6 through isothermal titration calorimetry (ITC). The ITC data revealed that AcH4-21 binds SAH-TbPRMT6 with a *K*_d_ of 45 µM, whereas no binding was observed for apo-TbPRMT6 (Figure 2C and Table S1), demonstrating that SAH binding greatly enhanced the affinity of the substrate peptide AcH4-21 to TbPRMT6. This enhancement in the affinity can be interpreted structurally. (i) The inward movement of R39 may create space for the accommodation of the guanidino group of the target arginine. (ii) The hydrogen bonding between D36 of helix Y and H318 of the ‘THW’ loop may stabilize helix Y, which constitutes the upper ridge of the substrate-binding groove, and fixes H318 in an appropriate position for target arginine binding. (iii) Hydrogen bonding with the main-chain amide of M144 and the side-chain atoms of R39 would stabilize the carboxyl group of E142 in a conformation favorable for target arginine binding and catalysis. The SAH-induced rearrangements may be a common feature because the residues involved are strictly conserved among type I PRMTs. Consistent with our structural observations, mutations of these residues completely abolish the activity toward the bovine histone H4 tail at Arg3 (Figure 2D), indicating that these residues are vital for the activity of TbPRMT6. Taken together, our structural observations and biochemical results suggest that SAH binding induces rearrangements of the active site that promote the substrate binding of TbPRMT6, thereby supporting an ordered mechanism in which a cofactor binds to the active site before the arginine substrate.

### Dimerization

**Figure 4:**
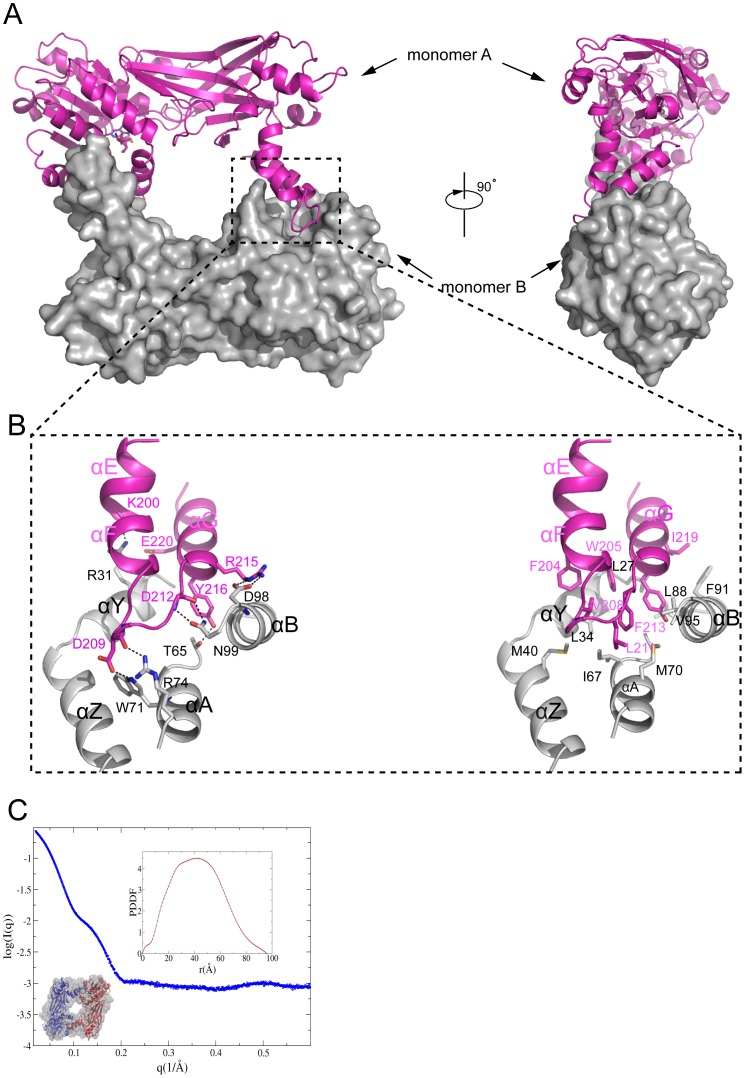
Dimerization of TbPRMT6. (A) Two views of the TbPRMT6 dimer Monomer A is shown as a cartoon colored in magenta, and monomer B is colored in gray and shown through a surface presentation (B) Dimerization interactions The left image represents the hydrogen bond interactions on the dimeric interface, and the right image represents the hydrophobic interactions (C) SAXS results of TbPRMT6_L_ The experimental SAXS curve of TbPRMT6 and the data points up to q  = 06 Å^−1^ are plotted The top-right inset is the PDDF calculated by GNOM, and the bottom-left inset is the DR model with the crystal structure superimposed with a TbPRMT6 dimer The R_g_ and D_max_ of TbPRMT6 are 328±01 Å and 959 Å, respectively The DR models were generated by GASBOR using the final χ against the raw SAXS data of 063 The X-ray structure of the TbPRMT6 dimer can be superimposed onto the DR models quite well, with an NSD of 109.

Dimerization is a feature that is strictly conserved in all known PRMTs. Similar to previously characterized PRMTs, TbPRMT6 also forms a homodimer in the crystal structure (Figure 1C and Figure 4A). To confirm this dimeric state in solution, we performed small-angle X-ray scattering assays. The molecular mass evaluated by SAXSMOW was approximately 85 kDa; because the molecular weight of TbPRMT6 is 41 kDa, this finding suggests that TbPRMT6 is found in a dimeric state in solution (Figure 4C).

The dimerization of TbPRMT6, which buries a solvent-accessible surface area of ∼1600 Å^2^, is mediated by helixes αE, αG, and αF (dimerization arm) of monomer A and helixes αY, αA, αB, and αC of the SAM-binding domain of monomer B (Figure 4B). The dimeric interface is essentially stabilized by extensive hydrophobic interactions and numerous hydrogen bonds. The hydrophobic interactions consist of a set of hydrophobic residues, including L27, L34, I67, M70, L88, and V95 in the SAM-binding domain of monomer B and F204, W205, V208, F213, and Y216 in the arm of monomer A (Figure 4B). The side chain of N99 of monomer B makes bifurcated hydrogen bonds with the backbone amide and carbonyl of D212 of monomer A, and this hydrogen bonding model is strictly conserved in PRMT dimerization interactions (Figure 3). In addition, R31, T65, W71, R74, and D98 of monomer B form hydrogen bonds with K200, Y216, D209, D209, and R215 of monomer A (Figure 4B), respectively. In addition to hydrogen bonding with K200, R31 of monomer B also forms a charge-charge interaction with E220 of monomer A (Figure 4B).

### Structural Comparison of TbPRM6 with Other PRMTs

To date, four type I PRMTs and a type II PRMT have been structurally characterized: rat PRMT1 (and its yeast homologue RMT1) [41]–[42], rat PRMT3 [43], rat CARM1/PRMT4 [39], [44], AbPRMT10 [45] and PRMT5 [46]–[48]. TbPRMT6 only shares 30% amino acid identity with PRMT1, which is lower than that of RMT1 (49%), PRMT3 (49%), CARM1 (34%), and PRMT10 (33%). Secondary structure-based sequence alignment reveals unique sequence features of TbPRMT6: four stretches of insertion (I: residues 114–127, II: residues 168–175, III: residues 275–289, and IV: residues 327–333) and a truncated C terminus (Figure 3, the four insertions are highlighted in purple frames). It is worth to note that the structures of these regions are strictly conserved among PRMT1, PRMT3 and PRMT4 (Figure S1).

**Figure 5:**
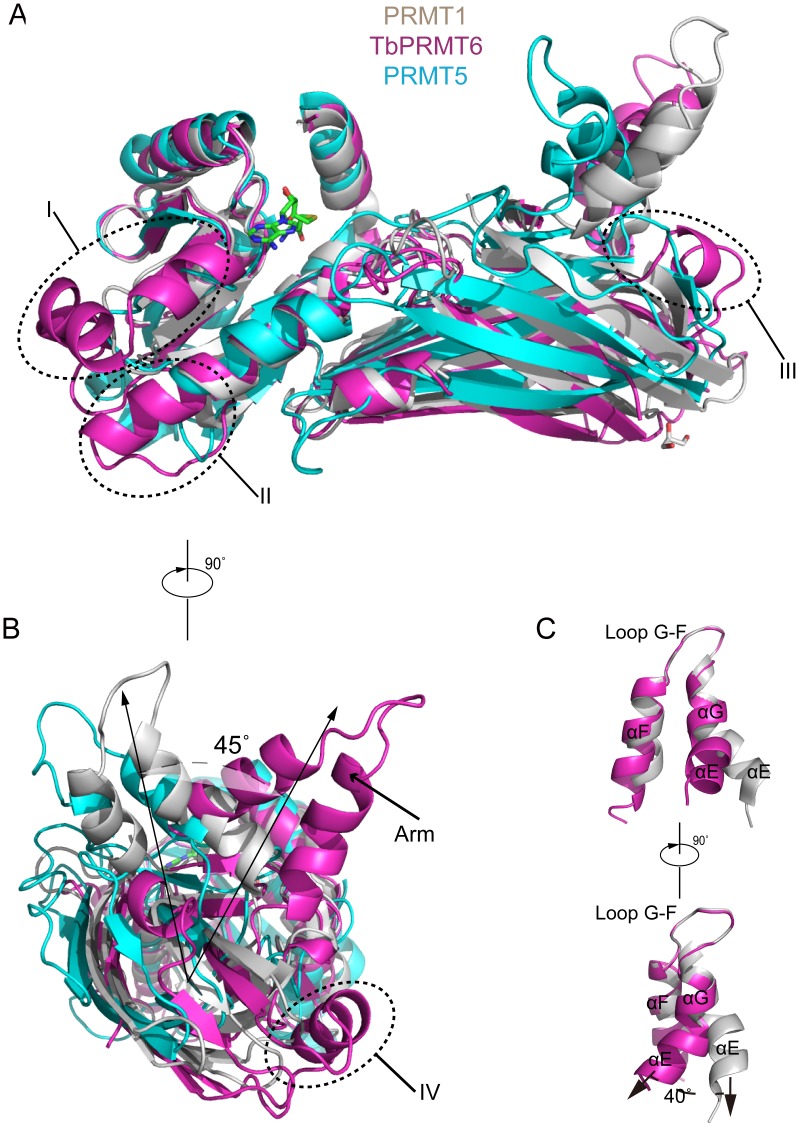
Structural comparison of TbPRMT6 with rat PRMT1 and human PRMT5. (A) and (B) Two views of the superposition of TbPRMT6 (magenta) with rat PRMT1 (gray, PDB code: 1OR8) and human PRMT5 (cyan, PDB code: 4GQB) The structural differences between TbPRMT6 and rat PRMT1 are labeled with arrows (C) Two views of the superposition of the dimerization arms of TbPRMT6 (magenta) and rat PRMT1 (gray).

We used PRMT1 as a representative of other type I PRMTs, and PRMT5 as a representative of type II PRMTs. Structural superposition of TbPRMT6 to PRMT1 and PRMT5 showed numerous marked differences (Figure 5A). A significant difference is the segment between strands β3 and β4. This segment is a loop in PRMT1, PRMT5 and other type I PRMTs (Figure 5A) but has a 14-residue insertion (insertion I) in TbPRMT6 and folds into three short helixes (αC, αC’, and αC”), which are packed against helix αD and strands β1, β2, and β3 (Figure 5B and Figure S2). Insertion II is found between helix αD and strand β5 and creates an extension of helix αD and a longer following loop (Figure 5A). Insertion III folds into helix αJ and interacts with helix αI and loop β12’-β13 (Figure 5A). The segment corresponding to strand β12 of PRMT1 is disrupted into two shorter β strands (β12 and β12’) by insertion IV, which folds into helix αK (Figure 5A). Interestingly, this segment in PRMT5 is composed of two β strands and a connecting loop (Figure 5A). Another significant diversity can be observed in the arm of TbPRMT6 (Figure 5B). The composition of the arms of TbPRMT6 and PRMT1 is identical: these contain three helixes (αE, αG, and αF) and one loop (loop F–G) (Figure 5C). The conformations of helixes αG and αF and loop F-G of the two arms are similar, but helix αE of TbPRMT6 adopts an orientation that is quite different from that of the corresponding helix in PRMT1, i.e., it twists approximately 40° toward helix αE of PRMT1 (Figure 5C). In comparison with PRMT1, we found that the entire dimerization arm twists approximately 45° toward the arm of PRMT1 (Figure 5B). TbPRMT6 also lacks strand β15, which exists in PRMT5 and other type I PRMT structures (Figure 5A and Figure 3). Without the packing from strand β14, helix αG in the dimerized structure of TbPRMT6 is markedly more flexible than in other type I PRMTs (Figure S3). PRMT6 and PRMT7 appear to be the only two PRMTs that exist in kinetoplastid parasites(*T. brucei*, *T. cruzi*, and *L. major*) but not other parasitic protozoa [22]. Sequence alignments indicate that the four stretches of insertion and the absence of strand β15 are conserved among PRMT6 orthologs in kinetoplastids (Figure S4) but not conserved in HsPRMT6 (Figure 3).

### TbPRMT6 does not Methylate *T. brucei* Histone Peptides in vitro

**Figure 6:**
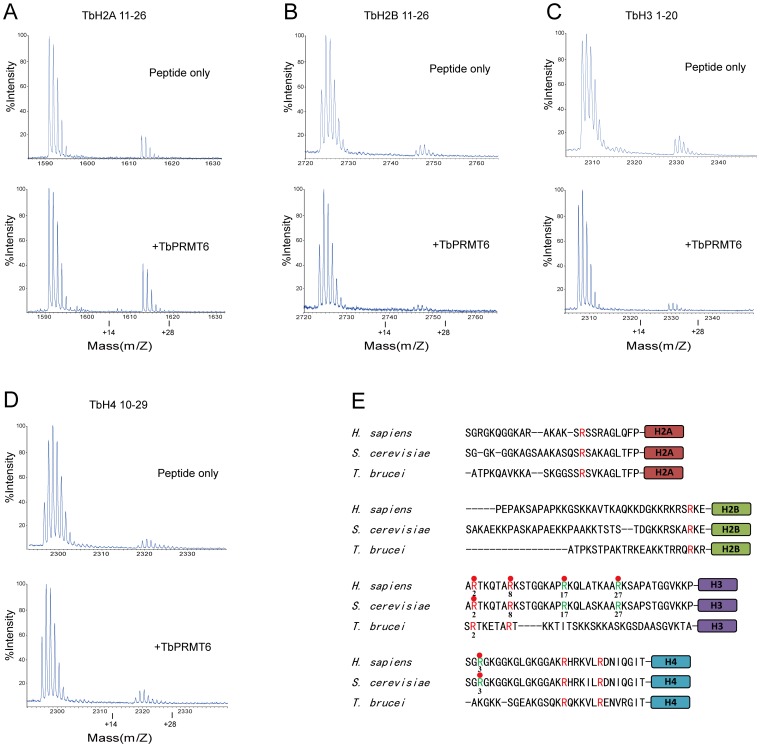
TbPRMT6 does not methylate *T brucei* histone tails. A–E: Two micrograms of histone tail peptides were incubated with 3 µg of TbPRMT6 in the presence of SAM and then subjected to MALDI-TOF MS analysis F: Comparison of N terminal tails of yeast, *T brucei*, and human histones The arginine residues conserved among all the three species are shown in red, and the arginine residues that are conserved in yeast and human but not conserved in *T brucei* are colored in green Those arginine residues that subjected to methylation in yeast or mammal are labeled with red balls.

The arginine residues located in the N-terminal tails of mammal histones are subjected to methylation by PRMTs [2], but the arginine methylation of *T. brucei* histones has not been reported [49]. The only known *in intro* substrates of TbPRMT6 are bovine histone H3, H4, and itself [19]. It has been reported that TbPRMT6 interacts with *T. brucei* histones *in vivo* [19]. To investigate whether *T. brucei* histone tails can act as TbPRMT6 substrates *in vitro*, we incubated TbPRMT6 with peptides derived from the N-terminal tails of TbH2A (residues 1–23), TbH2B (residues 11–26), TbH3 (residues 1–20), and TbH4 (residues 10–29), which covered all arginine residues in the N-terminal tails of *T. brucei* histones, and then subjected the reaction mixture to mass spectroscopy (Figure 6). Unfortunately, we did not observe any monomethylated products with an increased molecular weight (MW) of 14 Da or dimethylated products with an increased MW of 28 Da for the TbH2A, TbH2B, TbH3, and TbH4 peptides (Figure 6A-6D). These results suggest that TbPRMT6 cannot methylate the N-terminal tails of T. brucei histones *in vitro*.

The inability of TbPRMT6 to methylate *T. brucei* histone N-terminal tails may be due to the divergences between *T. brucei* histones and mammal histones. The amino acid sequences of histone N-terminal tails are extremely conserved, from yeast to mammals. However, the N-terminal tails of *T. brucei* histones are quite different from that of yeast and mammal histones (Figure 6E). Previous studies have shown that H3R2 and H4R3 in yeast histones, and H2AR3, H3R2, H3R8, H3R17, H3R27 and H4R3 in mammal histones are subjected to methylation [3]. Among these arginine residues, only H3R2 and H3R8 are conserved in *T. brucei* histones (Figure 6E). The previous work showed that TbPRMT6 methylate bovine histone H4 *in vitro* [19], and our result also showed that TbPRMT6 can methylate bovine histone H4R3 (Figure 2D). Arg3 is strictly conserved in yeast and mammal histone H4 but not exists in *T. brucei* histone H4 (Figure 6E), which may lead to that TbPRMT6 display no activity toward *T. brucei* histone H4 N-terminal tail. Beside bovine histone H4, TbPRMT6 were shown to methylate bovine histone H3 *in vitro* [19]. The first five residues of yeast and mammal histone H3 are ‘ARTKQ’, while the corresponding sequence in *T. brucei* histone H3 is ‘SRTKE’ (Figure 6E). The divergences in the flanking residues of Arg 2 may result in that TbPRMT6 is unable to target *T.brucei* histone H3 tail as to methylate bovine histone H3. One possibility is that TbPRMT6 lost activity towards *T. brucei* histone tails due to their evolutionary divergences, but still retain the activity towards bovine histones, which are strictly conserved in most eukaryotic species. Consistent with our result, a recent global proteomic analysis has detected 1332 methylarginines in 676 *T. brucei* proteins, but failed to indentify methylargnine residues in *T. brucei* histones [50]. Thrillingly, targeted studies to detect methylarginine in *T. brucei* histones have been underway in Read’s laboratory [50]. We are looking forward the result to resolve the question that whether *T. brucei* histones are subjected to arginine methylation.

## Supporting Information

Figure S1 Structural comparison of TbPRMT6 with other type I PRMTs. Rat PRMT1, rat PRMT3, rat CARM1, AtPRMT10 and TbPRMT6 are shown in cartoon colored in cyan, yellow, wheat, gray and magentas, respectively. Unique structural features are labeled with arrows.
(TIF)

Figure S2 Interaction of helixes αC, αC’ and αC” with strands β1, β2, β3, β4 and helix αD. The interaction is mainly stabilized by hydrophobic residues I115, I116, V120, V124, H127, L131 of helixes αC-αC” (in magentas), F81, I83 of strand β2, L108 of strand β3 and F163, F164, V171 of strand β3 (in cyan). K130 of helix αC” also makes a hydrogen bond with T106 of strand β3.
(TIF)

Figure S3 The B factor putty representations of dimer of TbPRMT6, PRMT1, PRMT3, CARM1 and AbPRMT10. Regions of higher B-factor are shown with larger diameter and colored in red. The helix αG is indicated with arrows respectively.
(TIF)

Figure S4 Sequence alignment of TbPRMT6 to parologs in *Trypanosoma cruzi*, and *Leishmania major*. The secondary structural elements of TbPRMT6 are shown on the top of the sequence. The color of secondary structural elements is as that of Figure 1B. Residues conserve among the three enzymes are highlighted in black. The four stretches of insertion in Figure 3 are bracketed with red dash frame and labeled.
(TIF)

Table S1 Thermodynamic parameters of ITC titrations.
(DOCX)
