# Supplementary figures and images for: Crystal Structure of Arginine Methyltransferase 6 from Trypanosoma brucei
Source: PLoS One. 2014 Feb 3;9(2):e87267. doi: 10.1371/journal.pone.0087267 (PMC3911951; doi:10.1371/journal.pone.0087267)

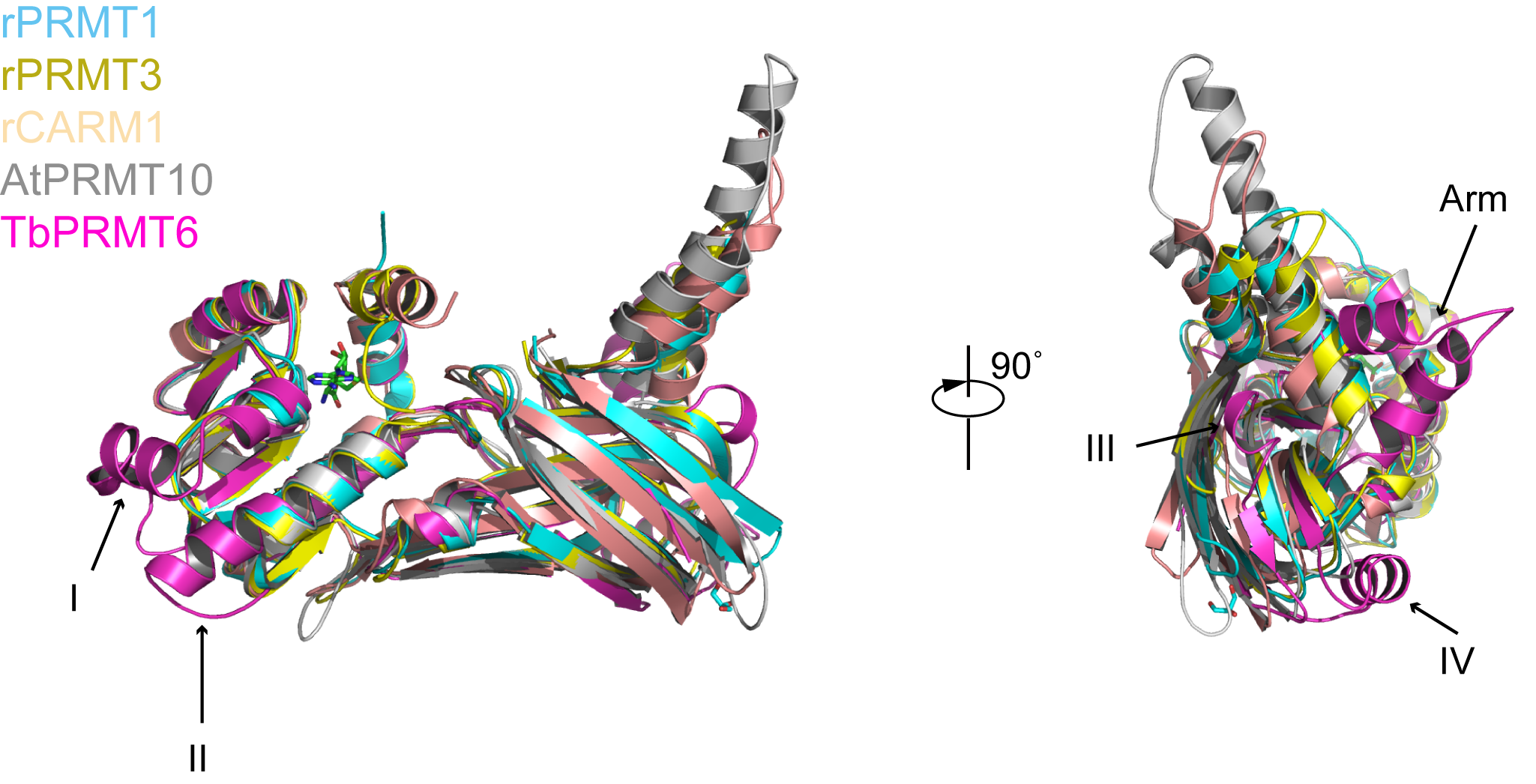

Supplement: Figure S1 — Structural comparison of TbPRMT6 with other type I PRMTs. Rat PRMT1, rat PRMT3, rat CARM1, AtPRMT10 and TbPRMT6 are shown in cartoon colored in cyan, yellow, wheat, gray and magentas, respectively. Unique structural features are labeled with arrows. (TIF) [file pone.0087267.s001.tif]

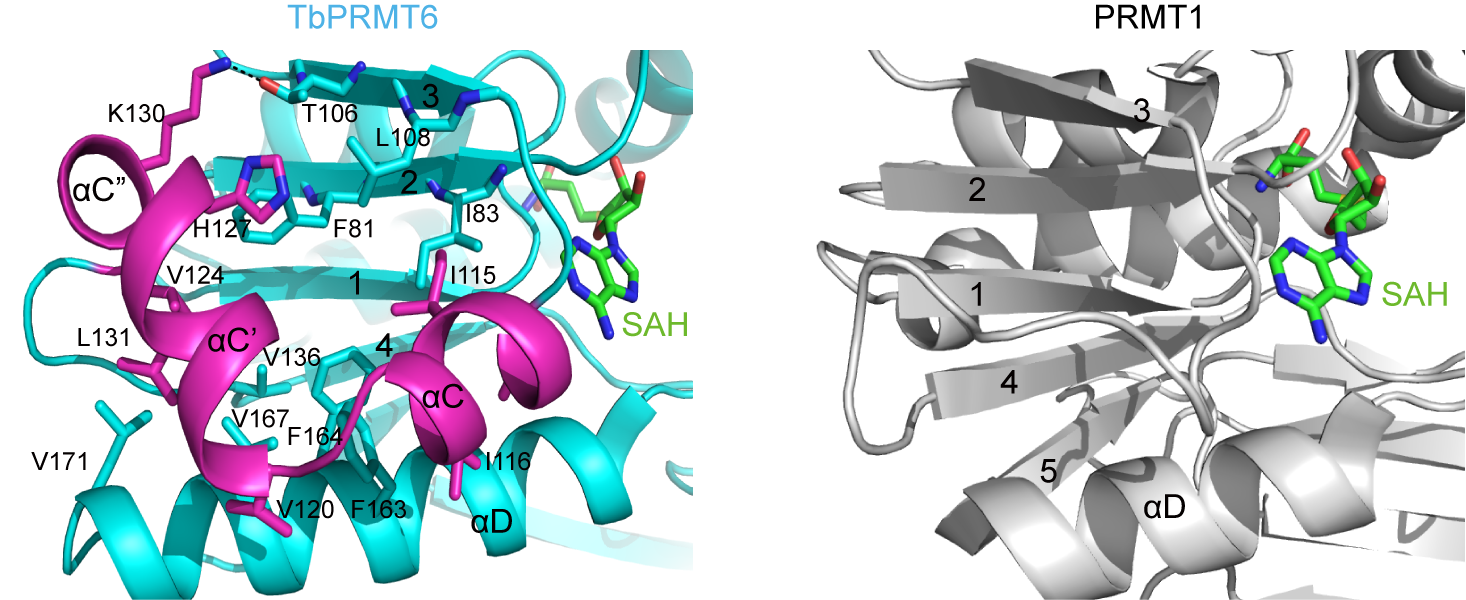

Supplement: Figure S2 — Interaction of helixes αC, αC’ and αC” with strands β1, β2, β3, β4 and helix αD. The interaction is mainly stabilized by hydrophobic residues I115, I116, V120, V124, H127, L131 of helixes αC-αC” (in magentas), F81, I83 of strand β2, L108 of strand β3 and F163, F164, V171 of strand β3 (in cyan). K130 of helix αC” also makes a hydrogen bond with T106 of strand β3. (TIF) [file pone.0087267.s002.tif]

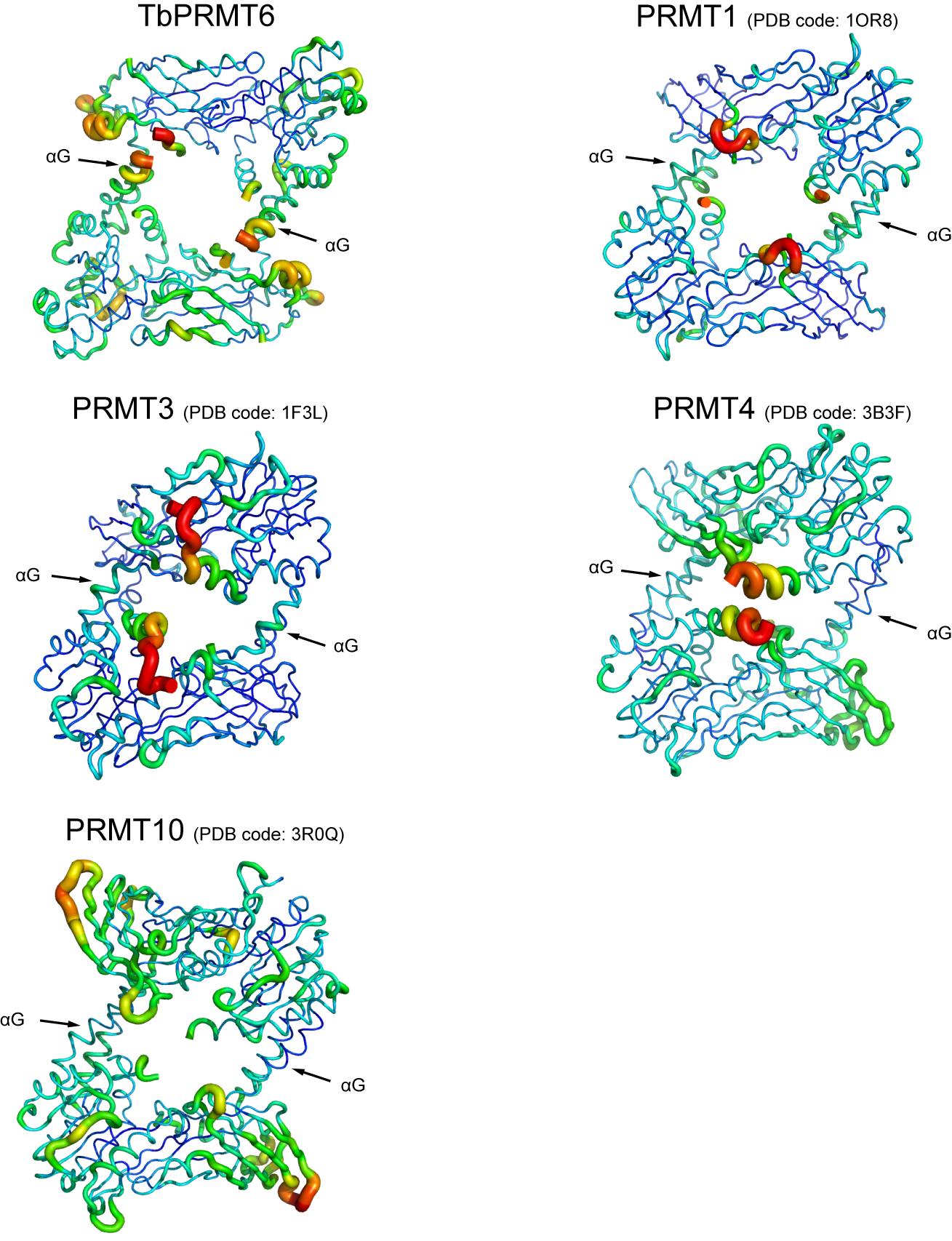

Supplement: Figure S3 — The B factor putty representations of dimer of TbPRMT6, PRMT1, PRMT3, CARM1 and AbPRMT10. Regions of higher B-factor are shown with larger diameter and colored in red. The helix αG is indicated with arrows respectively. (TIF) [file pone.0087267.s003.tif]

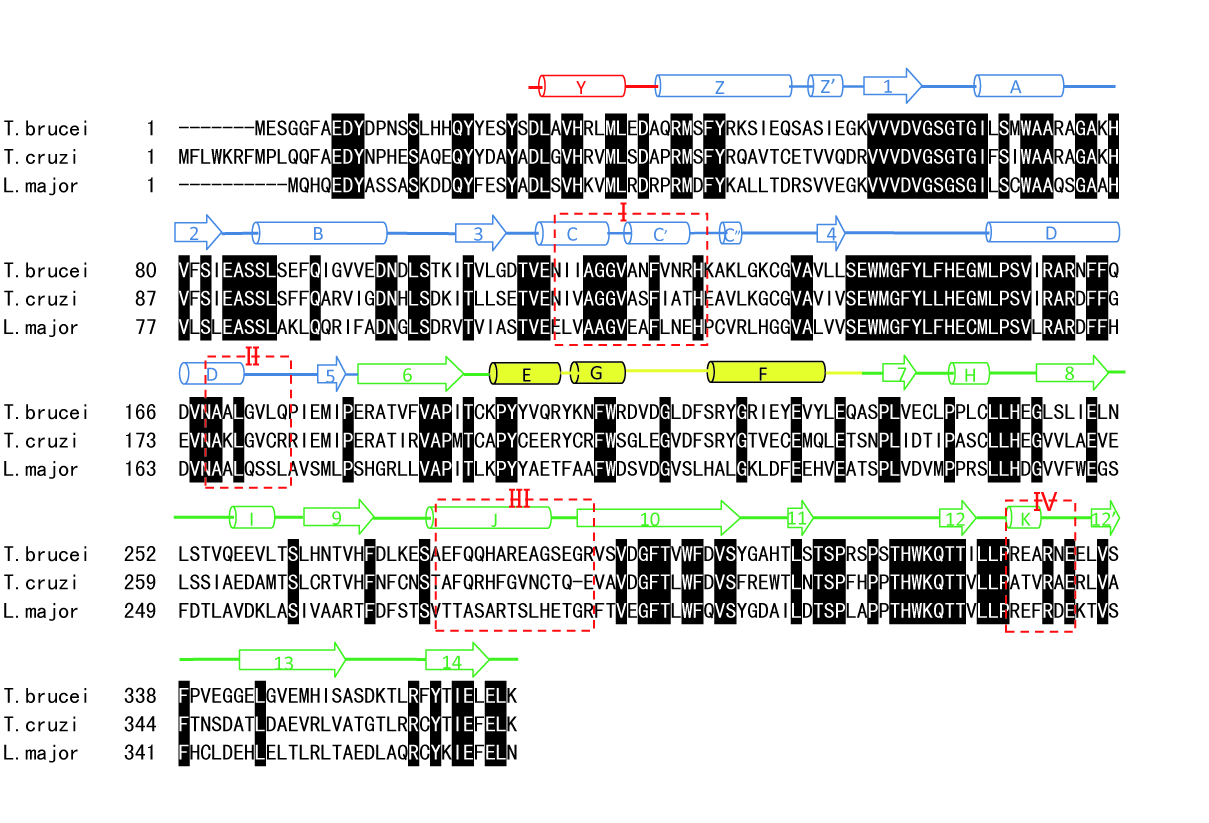

Supplement: Figure S4 — Sequence alignment of TbPRMT6 to parologs in Trypanosoma cruzi, and Leishmania major. The secondary structural elements of TbPRMT6 are shown on the top of the sequence. The color of secondary structural elements is as that of Figure 1B. Residues conserve among the three enzymes are highlighted in black. The four stretches of insertion in Figure 3 are bracketed with red dash frame and labeled. (TIF) [file pone.0087267.s004.tif]
