# Supplementary material for: Crystal Structure of Arginine Methyltransferase 6 from Trypanosoma brucei
Source: PLoS One. 2014 Feb 3;9(2):e87267. doi: 10.1371/journal.pone.0087267 (PMC3911951; doi:10.1371/journal.pone.0087267)
Supplement: Table S1 — Thermodynamic parameters of ITC titrations. (DOCX) [file pone.0087267.s005.docx]

Table S1. Thermodynamic parameters of ITC titrations

| **Proteins** | **Peptides** | **ΔH**  **kcal/mol** | **ΔS**  **cal/mol/K** | **K_d_**  **μM** | **N** |
| --- | --- | --- | --- | --- | --- |
| **TbPRMT6-SAH** | AcH4-21 | -0.31±0.01 | 18.9 | 45±4.5 | 1.79 |
